# B cell rich meningeal inflammation associates with increased spinal cord pathology in multiple sclerosis

**DOI:** 10.1111/bpa.12841

**Published:** 2020-04-26

**Authors:** Camilla Reali, Roberta Magliozzi, Federico Roncaroli, Richard Nicholas, Owain W. Howell, Richard Reynolds

**Affiliations:** ^1^ Department of Brain Sciences Faculty of Medicine Imperial College London UK; ^2^ Merck Healthcare KGaA Darmstadt Germany; ^3^ Department of Neuroscience, Biomedicine and Movement University of Verona Verona Italy; ^4^ Division of Neuroscience and Experimental Psychology Faculty of Biology, Medicine and Health University of Manchester Manchester UK; ^5^ Manchester Academic Health Science Centre Manchester UK; ^6^ Institute for Life Sciences Swansea University Medical School Swansea UK

**Keywords:** axon loss, B‐cell follicle, demyelination, lymphoid‐like structures

## Abstract

Increased inflammation in the cerebral meninges is associated with extensive subpial cortical grey matter pathology in the forebrain and a more severe disease course in a substantial proportion of secondary progressive multiple sclerosis (SPMS) cases. It is not known whether this relationship extends to spinal cord pathology. We assessed the contribution of meningeal and parenchymal immune infiltrates to spinal cord pathology in SPMS cases characterized in the presence (F+) or absence (F−) of lymphoid‐like structures in the forebrain meninges. Transverse cryosections of cervical, thoracic and lumbar cord of 22 SPMS and five control cases were analyzed for CD20+ B cells, CD4+ and CD8+ T cells, microglia/macrophages (IBA‐1+), demyelination (myelin oligodendrocyte glycoprotein+) and axon density (neurofilament‐H+). Lymphoid‐like structures containing follicular dendritic cell networks and dividing B cells were seen in the spinal meninges of 3 out of 11 F+ SPMS cases. CD4+ and CD20+ cell counts were increased in F+ SPMS compared to F− SPMS and controls, whilst axon loss was greatest in motor and sensory tracts of the F+ SPMS cases (*P* < 0.01). The density of CD20+ B cells of the spinal leptomeninges correlated with CD4+ T cells and total B and T cells of the meninges; with the density of white matter perivascular CD20+ and CD4+ lymphocytes (*P* < 0.05); with white matter lesion area (*P* < 0.05); and the extent of axon loss (*P* < 0.05) in F+ SPMS cases only. We show that the presence of lymphoid‐like structures in the forebrain is associated with a profound spinal cord pathology and local B cell rich meningeal inflammation associates with the extent of cord pathology. Our work supports a principal role for B cells in sustaining inflammation and tissue injury throughout the CNS in the progressive disease stage.

## Introduction

Multiple sclerosis (MS) is a demyelinating and neurodegenerative disease of the brain and spinal cord characterized by adaptive and innate immune system activation and a profound accumulation of irreversible neurological disability. Monoclonal antibody therapies targeting the B cell antigen CD20 are beneficial in the early relapsing‐remitting stage and in active primary progressive (PPMS) patients, highlighting the prominent role B cells play in the disease process (23, 29, 43, 45). Aggregates of B lymphocytes, which sometimes resemble ectopic B cell rich lymphoid‐like structures, are seen in the forebrain leptomeninges in early (acute and relapsing‐remitting MS) and late disease (secondary progressive MS; SPMS) and closely associated with the sites of underlying demyelination, microglia/macrophage activation and neurodegeneration (6, 22, 25, 35, 40, 49). It is unclear if lymphoid‐like structures exist in other leptomeningeal compartments and if they ever form adjacent to sites of white matter pathology. Given the critical involvement of the pathology of the spinal cord to the diagnosis and prognosis of MS, we thought it pertinent to explore these questions further.

Demyelination is extensive in the white and grey matter of the spinal cord (19). The extent of demyelination in part reflects atrophy of the cord and both whole cord and grey matter‐specific measures of atrophy correlate with clinical disease severity. Neurodegeneration in the cord undoubtedly contributes to both motor and sensory disability (19, 33, 48) and there is widespread synaptic, neuronal and axonal loss (14, 16, 20, 52, 57), which is seen in acute MS and could be the result of both local and distal pathological events (20, 48, 52).

Leptomeningeal inflammation, characterized by increased numbers of circulating and long‐lived B cells, plasma cells and large numbers of CD4+ and CD8+ T cells, is seen in a substantial proportion of MS cases, where it may play an important role in initiating and/or modulating demyelinating pathology (10). Clones of related B cells are shared between pathological sites in the brain (34) and are efficiently able to activate T cells and myeloid cells, and act as antigen‐presenting cells (29). Subsets of memory B cells are a source of soluble inflammatory mediators, which are toxic to neurons and oligodendrocytes in vitro (31, 32). Studies focussed on the spinal cord have shown a prominent inflammation of the surrounding meninges, which is related to the extent of forebrain inflammation (58), whereby T cell infiltrates associate with parenchymal inflammation, demyelination and diffuse axonal loss (2, 12). However, the contribution of B cells to spinal cord pathology has hitherto not been assessed in detail.

Our previous work has shown an involvement of meningeal inflammation, in particular the formation of ectopic lymphoid‐like structures in the sulci, in the pathogenesis of cerebral and cerebellar cortical grey matter lesions in patients with acute and chronic progressive MS. Approximately 40% of all cases of SPMS harbored at least one (some with many) detectable lymphoid‐like structure in their forebrain meninges (6, 11, 24, 39, 40, 54). These findings support the hypothesis that meningeal inflammation gives rise to an increased inflammatory milieu in the CSF space, in particular the subarachnoid space, which might in part drive the formation of grey matter lesions and an accompanying increased severity of clinical disease in some patients (38). The cytotoxic inflammatory milieu gives rise to alterations to the glial limitans and subsequent activation of microglia and neurodegeneration (18, 39). Whether the same pathogenetic mechanisms may be operating in the spinal cord and contribute to the more severe pathology in cases that exhibit ectopic lymphoid‐like structures is unknown.

The aim of this study was to investigate the detailed cellular immunopathology of the spinal cord and meninges from cases of SPMS already characterized in terms of the presence or absence of detectable lymphoid‐like structures in the forebrain leptomeninges. This work gives an insight into the CNS‐wide extent of compartmentalized inflammation and diffuse meningeal infiltrates seen in some cases of SPMS, whilst confirming a key association between meningeal CD20+ B cells and the underlying demyelinating and neurodegenerative pathology.

## Materials and Methods

### Tissue samples

Post‐mortem spinal cord tissue blocks from 22 cases of SPMS and from five non‐neurological control cases were provided by the UK MS Tissue Bank at Imperial College. No cases of neuromyelitis optica were included in this study. All tissues were collected with fully informed consent via a prospective donor scheme with ethical approval by the National Research Ethics Committee (08/MRE09/31). Relevant clinical and demographic data are shown in Table 1 and included information on the number and date of spinal relapses. All tissue blocks were paraformaldehyde fixed (mean fixation time = 17 ± 6 days), cryoprotected in sucrose solution and frozen in isopentane on a bed of dry ice. Transverse sections of 10µm thickness were cut on a cryostat and stored at −80°C until required. To better understand the relationship between meningeal lymphoid‐like structures and the extent of the spinal cord pathology, we analyzed the blocks of spinal cord from cases previously analyzed in work describing meningeal inflammation and forebrain pathology (24, 39). We examined sections from three spinal cord tissue blocks (taken at the level of the cervical, thoracic and lumbar cord) per case, from 11 SPMS cases with lymphoid‐like structures in the cerebral meninges (F+ SPMS), and 11 cases without detectable lymphoid‐like structures (F− SPMS; Table 1).

**Table 1:** Clinical information of multiple sclerosis and control cases.

Case	Age at death	Sex	Disease duration	Age at Onset	Spinal relapse (Y/N)	PMD (hours)	Cause of death	F+ blocks/total forebrain blocks
F+ SPMS								
MS46	40	M	23	17	Y	18	Multiple sclerosis	3/11
MS79	49	F	24	25	?	7	Bronchopneumonia	5/12
MS85	59	F	35	24	?	27	Cerebrovascular Accident	2/11
MS92	37	F	17	20	N	26	Multiple sclerosis	7/12
MS154	34	F	11	22	Y	12	Pneumonia	4/12
MS160	44	F	16	28	Y	18	Bronchopneumonia	4/12
MS234	39	F	39	24	Y	15	Pneumonia	4/12
MS256	53	F	24	29	Y	21	Aspiration pneumonia	2/11
MS317	48	F	29	19	Y	21	Aspiration pneumonia	5/16
MS330	59	F	39	20	Y	21	Pneumonia	7/22
MS342	35	F	5	30	N	9	Multiple sclerosis	2/14
F− SPMS								
MS3	55	M	21	34	Y	44	Urinary tract infection	0
MS21	58	M	41	17	N	16	Urinary tract infection	0
MS71	78	F	42	36	Y	5	Carcinoma of the bronchus	0
MS74	64	F	28	36	Y	7	Bronchopneumonia	0
MS101	50	M	31	19	Y	24	Bronchopneumonia	0
MS104	53	M	11	42	Y	12	Urinary tract infection	0
MS114	52	F	15	37	N	12	Bronchopneumonia	0
MS127	51	M	23	28	N	21	Bronchopneumonia	0
MS139	62	F	22	40	Y	9	Bronchopneumonia	0
MS340	53	F	19	34	N	17	Sepsis	0
MS341	52	F	22	30	Y	8	Aspiration pneumonia	0
Control								
C07	85	F	N/A	N/A	N/A	N/A	Cancer of the oesophagus	N/A
C09	84	F	N/A	N/A	N/A	N/A	Cerebrovascular disease	N/A
C32	88	M	N/A	N/A	N/A	N/A	Prostate cancer	N/A
C41	54	M	N/A	N/A	N/A	N/A	Lung cancer	N/A
C45	77	M	N/A	N/A	N/A	N/A	Cardiopulmonary degeneration	N/A

Abbreviations: ? = not known; F− SPMS = lymphoid‐like structure negative secondary progressive multiple sclerosis. F+ blocks/total forebrain blocks = frequency of lymphoid‐like structure positive blocks of total forebrain tissue blocks sampled [see Magliozzi *et al* (39) and Howell *et al* (24) for further information]. N/A = not applicable; F+ SPMS = lymphoid‐like structure positive secondary progressive multiple sclerosis.

### Immunohistochemistry

Sections were air‐dried, rehydrated in phosphate‐buffered saline (PBS, Sigma UK), and subjected to heat‐induced antigen retrieval as indicated (Table 2). Endogenous peroxidase activity was quenched with a solution of PBS/0.3% (v/v) H_2_O_2_ and non‐specific binding blocked by the addition of 10% normal sera in PBS. Primary antibodies (Table 2) were prepared in PBS, 0.2% Triton X‐100 and 1% normal sera, and added to slides for an overnight incubation at 4 °C. Bound primary antibody was detected by a two‐step procedure by first incubating with an appropriate biotinylated secondary antibody (Vector Labs, UK) followed by the addition of the avidin‐biotin horseradish peroxidase complex (ABC VECTASTAIN Elite kit; Vector) and 3,3′‐diaminobenzidine (DAB) (IMMPACT DAB, Vector) as chromogen. Negative controls were included in each experiment and all sections from all cases were stained together in the same experimental run. Sections were counterstained in Haematoxylin, dehydrated, cleared in xylene and coverslipped in Depex Polystyrene (DPX; Sigma).

**Table 2:** Primary antibodies for immunohistochemistry/immunofluorescence.

Antigen	Cell specificity	Clone	Source
CD20^†^, ^¶^	B lymphocytes	L26	ThermoFischer
CD35^‡^, ^¶^	Follicular dendritic cells	Ber/MAC/DRC	Agilent Dako
CD8^†^, ^¶^	Cytotoxic T cells	Rabbit pc	ThermoFisher
CD8^†^, ^¶^	Cytotoxic T cells	C8/144B	Agilent Dako
CD4^†^, ^¶^	T helper cells	BC/1F6	Abcam
Ig‐A, ‐G, ‐M (FITC conjugated)^¶^	Plasma cells	Rabbit pc	Agilent Dako
Ki67^†^	Proliferating cells	Rabbit polyclonal	Novacastra Labs
MOG^§^	Myelin and oligodendrocytes	Z12	*in house*
MBP^§^	Myelin basic protein	Rabbit pc	Agilent Dako
IBA‐1	Microglia/macrophages	Rabbit pc	Fujifilm Wako
Neurofilament heavy chain	Axons, dendrites	RT97	Chemicon/Merckmillipore
Neurofilament heavy chain	Neuron, axon, dendrite	Smi32	Chemicon/Merckmillipore

Antigen, cellular target and clone (for monoclonal antibodies) of antibodies used in this study. Antigen retrieval procedures.

^†^ Sodium citrate buffer pH 6.

^‡^ Dako target retrieval solution pH 9.

^§^ Permeabilization with cold methanol.

^¶^ Fixation with cold acetone.

### Immunofluorescence

Cryosections, prepared as above, were sequentially immunolabeled using different antibodies in combination (listed in Table 2). Bound primary antibody was detected using appropriate Cyanine 3 (Cy3)‐conjugated anti‐mouse IgG (1:500) and Alexa Fluor‐488‐conjugated anti‐rabbit IgG (1:500; Thermo Fisher, UK). All sections were counterstained with 4′,6‐diamidino‐2‐phenylindole (1:5000, DAPI, Sigma) and aqueous mounted in VECTASHIELD Aq (Vector).

### Quantification of demyelination

In order to determine the degree of demyelination in the spinal cord (n = 11 F+ and 11 F− SPMS), one section for each tissue block at each level was immunostained for myelin oligodendrocyte glycoprotein (MOG). Sections were scanned at 40x magnification using a Nikon Eclipse 80i microscope and the tiled images reconstructed using Image‐Pro Plus (Media Cybernetics. Inc, UK). The total and the demyelinated areas of each spinal cord section were manually outlined and measured using Image‐Pro Plus software and expressed relative to total white matter and/or total grey matter coronal area in that section.

### Quantification of lymphocytes

In order to analyze the distribution of B‐ and T‐lymphocytes in the spinal cord, we manually counted (under 200x magnification using a Leica DM2500 microscope) the number of anti‐ CD20+, CD4+ and CD8+ cells in the total spinal meninges, and total section white and grey matter perivascular space and parenchyma, respectively, in triplicate sections representing the three spinal cord levels sampled, per SPMS and control case. Lymphocyte counts were exhaustively conducted across the total section (n = 3), for each of the three anatomically matched sampled tissues, per case. Total counts per region of interest per section were recorded as the focal nature of the infiltrates and the sometimes very low incidence of lymphocytes meant that traditional field of view quantification would not have as accurately reported cord inflammation. The mean cell count per section, per case, was plotted and represented by a single point on the scatter graphs and groups (F+, F− SPMS and control), compared by Kruskal–Wallis test with Dunn's multiple comparison posttest (see “Statistical analysis” and legends).

### Quantification of axon loss

Digital images of heavy chain neurofilament H+ (by combining antibodies SMI32 and RT97 to non‐phosphorylated and phosphorylated epitopes, respectively) stained sections were used to calculate the number of axons in the anterior corticospinal tract, the lateral corticospinal tract and in the dorsal column from normal and demyelinated SPMS (see results for further information). Sections were scanned at 200x magnification (Nikon Eclipse 80i microscope) and the reconstructed whole section images handled in Image‐Pro Plus. Neurofilament H+ axons were counted automatically in the defined areas of interest following image thresholding to calculate axon density (neurofilament H+ axons/mm^2^) per region of interest per section, per cord level, per case. Relative axon loss was calculated by comparing axon density measures per SPMS case to the mean axon density value per region of interest in the control group.

### Quantification of microglia‐macrophages

The area of IBA‐1+ immunoreactivity was quantified as an index of microglia/macrophage density in the spinal cord. One IBA‐1/MOG double‐labeled section per spinal cord level (3 levels), from each F+ SPMS, F− SPMS and control case, was analyzed. Double staining for MOG delineated the grey‐ white matter boundary, allowing a comparison between microglia/macrophage densities in these regions. Images captured at 40x magnification were manually outlined to highlight white and grey matter and the relative area of IBA‐1+ immunoreactivity per area of white and grey matter, per spinal cord level, per case determined automatically following image thresholding.

### Statistical analysis

All data were assumed to be sampled from a non‐Gaussian distribution and non‐parametric analysis methods applied (44). The difference between two groups (for example, the density of axons in F+ SPMS vs. F− SPMS) was compared using the unpaired Mann‐Whitney test, whilst Kruskal–Wallis test with Dunn's multiple comparison posttest was used when comparing three or more groups (for example, when comparing the mean number of CD20+ cells per spinal meninges, between F+, F− SPMS and control. In this analysis each data point (n number) represents the mean number of cells from three sections, per cord level, per case). Spearman correlation was used to test for associations between groups and the Spearman *r* and *P* values reported in each instance. A two‐sided *P* value < 0.05 was considered significant (GraphPad PRISM v6).

## Results

**Figure 1:**
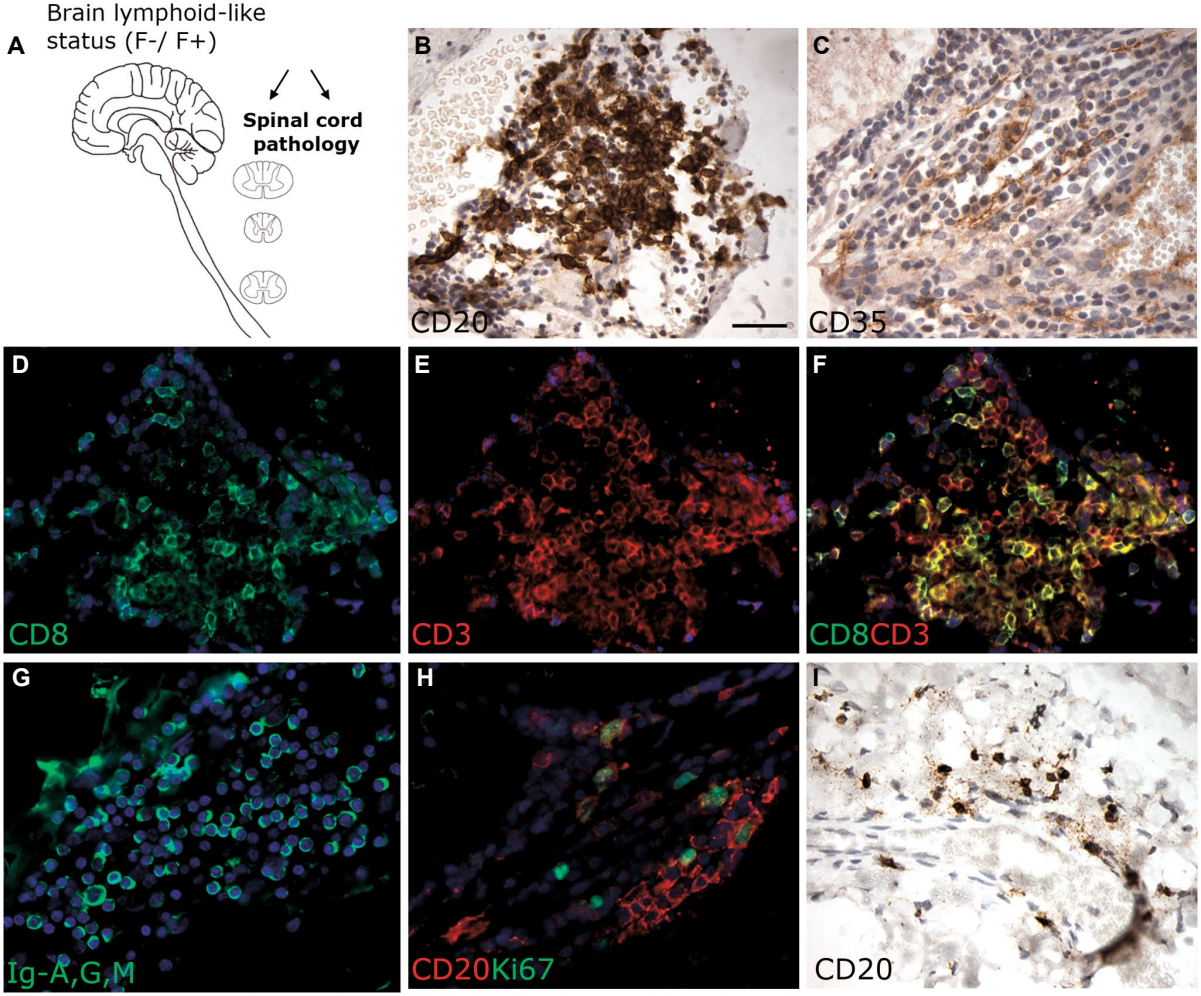
Characterization of ectopic lymphoid‐like structures in post‐mortem spinal cord tissue from SPMS cases previously characterized in the presence (F+) or absence (F−) of lymphoid‐like structures in the forebrain meninges (**A**). Immunostaining of serial spinal cord sections from an F+ SPMS case shows a meningeal lymphoid‐like structure comprising a dense aggregate of CD20+ B cells (**B**), a reticulum of CD35+ fibers consistent with the presence of follicular dendritic cells (**C**), CD3 and CD8+ T cells (**D**–**F**) and plasma cells labeled with an anti‐Ig‐G, ‐A, ‐M antibody (**G**). Proliferating B cells were identified by double immunofluorescence staining for CD20 (red) and Ki67 (green; **H**). CD20+ B cells were present in the meninges of F− SPMS cases but at lower densities and did not display any organised structure (**I**). Scale bar in B = 50µm applies to all images.

We aimed to investigate the association between lymphoid‐like structures, B and T cell infiltrates, demyelination and axon loss in the spinal cord of a cohort of SPMS cases that had been categorized based on the presence (F+ SPMS) or absence (F− SPMS) of B cell rich lymphoid‐like structures in the forebrain meninges (Figure 1A), to more fully understand the cellular mechanisms of progressive disease.

### Detection of lymphoid‐like structures in the spinal cord meninges

Meningeal lymphoid‐like structures with some of the features of tertiary lymphoid tissue were found in the spinal cord meninges of 3 out of the 11 F+ SPMS cases (MS79, MS92 and MS234; Table 1). Lymphoid‐like structures were identified in the presence of aggregates of CD20+ B cells, a reticulum of CD35+ follicular dendritic cells and by CD3+ and CD8+ T cells (Figure 1B–F). Lymphoid‐like structures also contained immunoglobulin+ plasma cells (Figure 1G) and evidence of proliferating (Ki67+) B cells (Figure 1H). These structures were found at all examined levels of the cord, with no dorsal, lateral or ventral preference. More often, a variable extent of B cell infiltration was noted in the spinal meninges in both F+ and F− SPMS cohorts (Figure 1I).

### Perivascular and meningeal inflammation in the SPMS spinal cord

**Figure 2:**
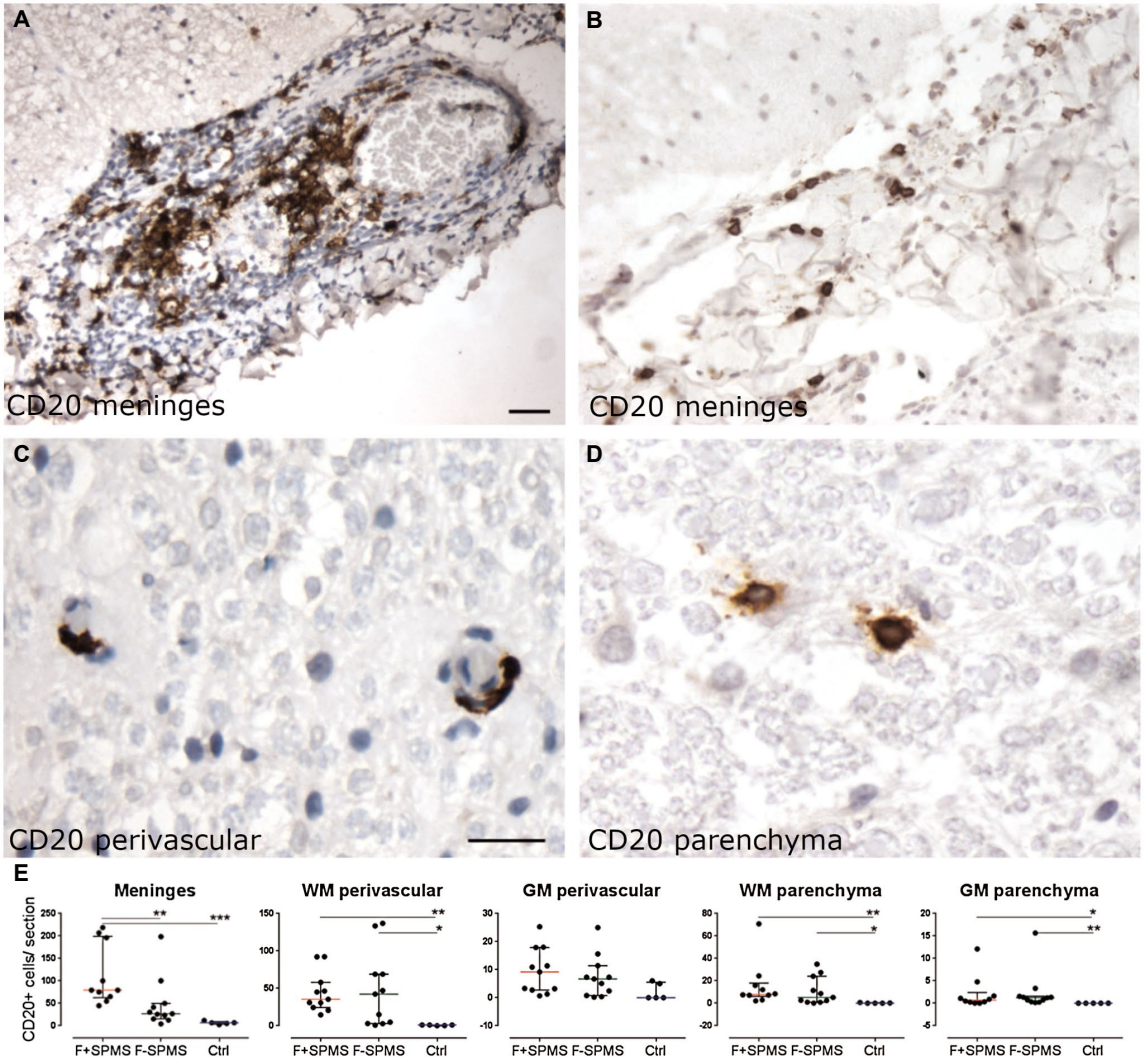
Quantifying meningeal, perivascular and parenchymal B cells in the spinal cord. Anti‐CD20 immunohistochemistry revealed moderate (**A**) to mild (**B**) infiltrates of B cells in the spinal meninges, alongside small numbers in the perivascular space and tissue parenchyma (**C**, **D**). The number of CD20+ B cells was greater in the F+ SPMS meninges compared to the F− SPMS and controls (**E**). The number of B cells was also increased in comparison to controls in perivascular and parenchymal tissues. Values represent total CD20+ cells per section, from three sections per case with median and interquartile range indicated. Kruskal–Wallis and Dunn's multiple comparison posttest. *P < 0.05, **P < 0.01. Scale bar: **A**, **B** = 40 µm; **C**, **D** = 30 µm. Ctrl, control; F+ SPMS, lymphoid‐like structure positive SPMS; F− SPMS, lymphoid‐like structure negative SPMS.

We quantified the extent of B cell infiltration in the spinal meninges and cord in F+ SPMS, F− SPMS and controls. Numbers of B cells varied greatly between the meninges, perivascular cuffs and tissue parenchyma (Figure 2A–D). There were more CD20+ B cells in the spinal meninges (cervical, thoracic and lumbar cord) of F+ SPMS in comparison to F− SPMS and controls (F+ SPMS = 79 ± 21, F− SPMS = 27 ± 17, Control = 7 ± 2 mean cells/section ± SEM, Figure 2E). The number of CD20+ cells was increased in perivascular cuffs of the white matter and in the tissue parenchyma of both white and grey matter, in F+ SPMS and F− SPMS, in comparison to controls (Figure 2E). In summary, cases categorized as F+ SPMS based on the finding of lymphoid‐like structures in the forebrain, were also the same cases that displayed elevated numbers of B cells in the spinal meninges [ie 9 of 11 cases of F+ SPMS had greater than the median number of B cells found in the SPMS spinal meninges (≥55 CD20+ cells; *P* = 0.0019; when comparing the frequency of cases with greater than the median number of B cells in the F+ and F− SPMS groups combined, Fisher's exact test)].

**Figure 3:**
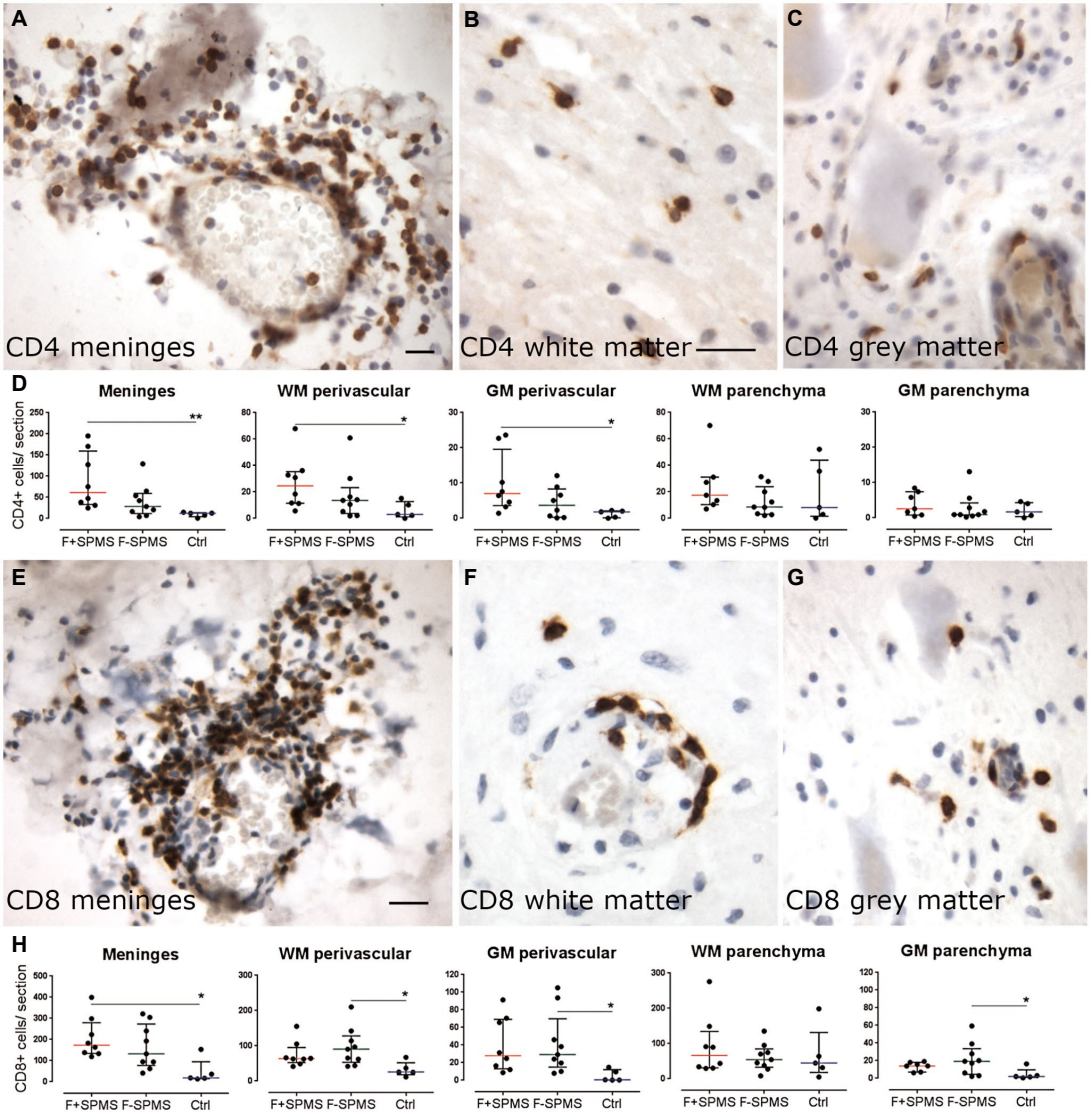
T cell infiltrates in the SPMS spinal cord. Immunohistochemical detection of CD4+ T cells in different compartments of the spinal cord of SPMS cases (**A**–**C**). The number of CD4+ T cells was greater in the meninges and in white and grey matter perivascular cuffs in F+ SPMS compared to controls (**D**). CD8+ T cells were occasionally seen as modest infiltrates in the meninges and at higher numbers than CD4+ T cells in perivascular spaces and parenchyma of the spinal cord white and grey matter (**E**–**G**). There was a large and variable extent of CD8+ infiltrates of the cord meninges, which differed between F+ SPMS and controls but not between F+ and F− SPMS (**H**). Scatter dot plot of total immunopositive cells per section, from three sections per case, with median and interquartile range indicated. Kruskal–Wallis and Dunn's multiple comparison posttest. *P < 0.05. Scale bar: 40 µm. Ctrl, control; F+ SPMS, lymphoid‐like structure positive SPMS; F− SPMS, lymphoid‐like structure negative SPMS.

CD4+ T cells were present as modest or sparse meningeal infiltrates (Figure 3A from a F+ SPMS case) and were sparsely distributed throughout the white and grey matter, both in the perivascular spaces and in the tissue (Figure 3B,C). CD4+ T cells were more numerous in F+ SPMS meninges, white and grey matter perivascular cuffs, in comparison to control tissue (Figure 3D). CD8+ T cells were also found in large numbers in the meninges of some SPMS cases and were typically found widely distributed throughout the white and grey matter of both F+ SPMS and F− SPMS, and in greater numbers than seen for CD4+ T cells or CD20+ B cells (Figure 3E–H; mean number ± SEM meningeal CD8 in F+ SPMS = 207 ± 34; CD4 = 89 ± 24; CD20 = 79 ± 21 cells/section). The approximate ratio of CD8:CD4:CD20 expressing cells was 2.5:1:1 and varied little between the different tissue compartments. Although the number of CD8+ T cells did not differ between F+ and F− SPMS cohorts, there were more CD8+ cells in the F+ SPMS meninges in comparison to controls (*P* < 0.05), and in F− SPMS white and grey matter perivascular cuffs compared with control (*P* < 0.05). There were also more CD8+ cells in grey matter parenchyma of F− SPMS in comparison to control tissue (*P* < 0.05, Figure 3H).

### Quantification of demyelination and axonal loss in the SPMS spinal cord

**Figure 4:**
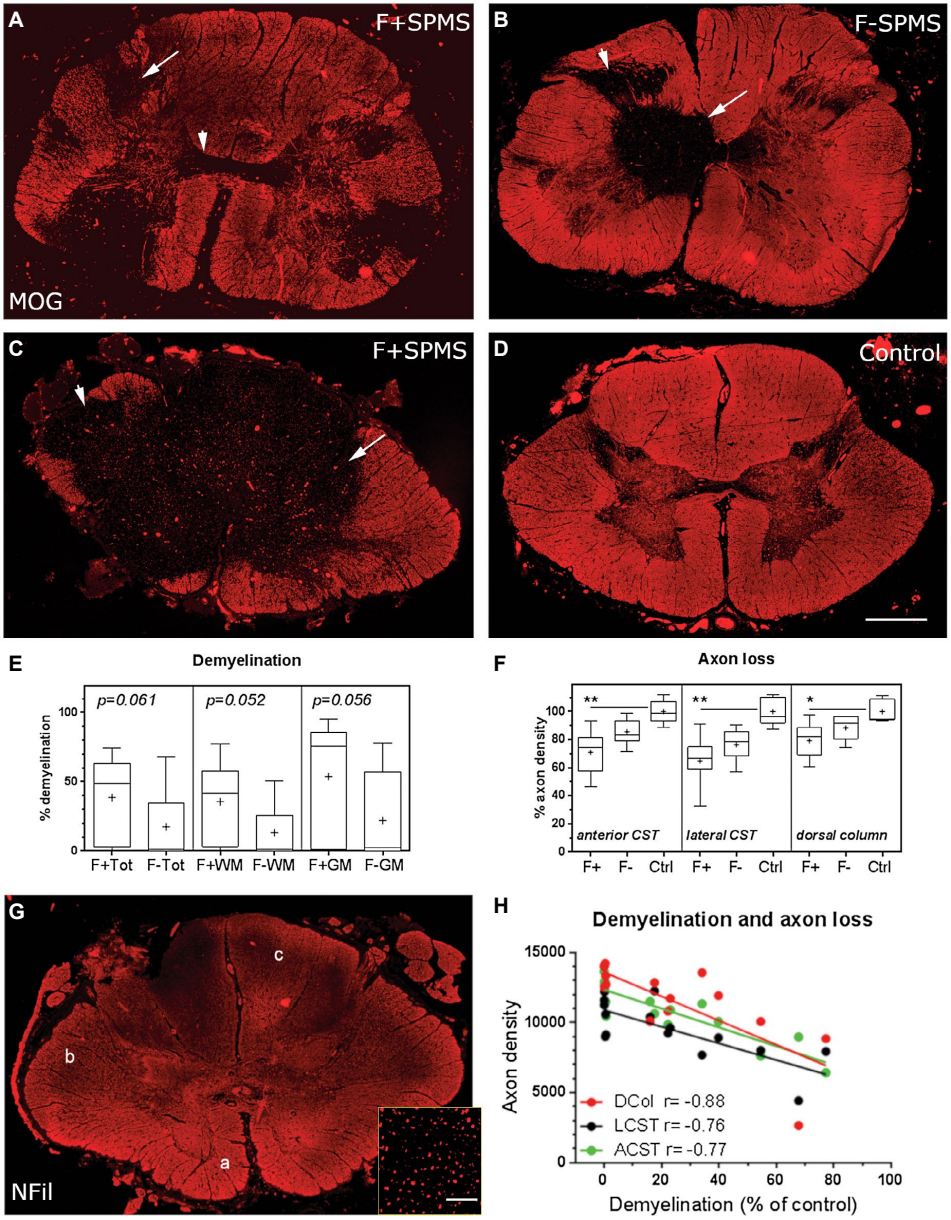
Demyelination and axon loss in the SPMS cord. Anti‐MOG immunostaining revealed the variable extent of demyelination in the F+ and F− SPMS spinal cord. Multiple discrete white and grey matter lesions or extensive confluent demyelination was seen in F+ SPMS, whereas lesions tended to be smaller in the F− SPMS cord (**A**–**C**; arrows). MOG+ myelin in a control case for comparison (**D**). The percentage of total (Tot), white matter (WM) and grey matter (GM) demyelination showed a trend to being greater in the F+ SPMS cohort in comparison to the F− SPMS cases (**E**). Axon density was determined by quantifying the number of neurofilament H+ (NFil) structures in the anterior (**a**) and lateral (b) corticospinal tracts (CST) and the dorsal column (**c**; **F**, **G**). Axon density relative to control counts were compared for each tract across the three sampled levels (cervical, thoracic and lumber cord) and expressed as percent axon loss. Axon density (%) was reduced in comparison to control axon counts in F+ SPMS cases at all sampled levels of the cord. Total axon count correlated with the relative area of white matter demyelination in F+ SPMS (*P* < 0.002 for each comparison; **H**). Box‐and‐whiskers plot showing the mean (+), median (line), interquartile range (box) and 5‐95 percentiles (whiskers) for each group. Differences between groups were tested by (E) the non‐parametric Mann‐Whitney test or (F) Kruskal–Wallis and Dunn's multiple comparison posttest. *P < 0.05, **P < 0.01. Correlation analysis by Spearman comparison. Scale bars (**A**–**D**, **G**) = 1 mm, inset 40 µm. Ctrl, control; F+, lymphoid‐like structure positive SPMS; F−, lymphoid‐like structure negative SPMS.

We assessed the demyelination of the spinal cord by anti‐MOG immunofluorescence across the sampled levels (Figure 4A–D). Total demyelinated lesion area per spinal cord ranged from zero to 74.5% of spinal cord cross‐section area (F+ SPMS total section mean lesion area = 39 ± 9%, F− SPMS = 17 ± 8%, *P* = 0.061) with areas of subpial white matter and grey matter around the central canal frequently affected. There was a trend toward a greater area of demyelinated white matter (36 ± 9%) and demyelinated grey matter (54 ± 13%) in F+ SPMS cases in comparison to F− SPMS (white matter, 13 ± 6%, *P* = 0.052; grey matter 22 ± 10%, *P* = 0.056; Figure 4E). When comparing the lesion area at each of the three sampled levels of the spinal cord, there was a trend to increasing lesion area in white and grey matter regions in F+ compared to F− SPMS (*P* < 0.1), but this was only significantly different in the cervical white and grey matter (white matter lesion area 44 ± 11% vs. 11 ± 6%, *P* = 0.016; grey matter lesion area 66 ± 14% vs. 24 ± 11%, *P* = 0.035; for F+ and F− SPMS, respectively).

Axon damage and loss is a prominent pathological feature of the MS spinal cord. We quantified the density of neurofilament‐H+ axons in both motor (anterior corticospinal tract and lateral corticospinal tract) and sensory (dorsal column) tracts across all three spinal cord levels in F+, F− SPMS and controls (Figure 4F, G). There was a reduction in the density of axons in F+ SPMS relative to control axon density at all sampled levels (data not shown). The relative density of axons in F− SPMS did not differ to either group at any sampled level of the spinal cord. When averaging the extent of axon loss relative to control across the three areas, F+ SPMS (69 ± 4.6%) differed to F− SPMS (83 ± 2%, *P* = 0.003) and to controls (100 ± 8.2%; *P* < 0.0001; comparing control relative axon density to either F+ SPMS or F− SPMS relative axon density; Figure 4F). There was a correlation between the extent of demyelination and the axon density in each tract across the sampled levels of the SPMS cord (Figure 4H, *P* < 0.002 in all comparisons). Correlations also existed in the sub‐analysis of the F+ SPMS and F− SPMS cohorts (comparing percent white matter demyelination with the density of F+ SPMS ACST (*r* = −0.91, *P* = 0.005), LCST (*r* = −0.74, *P* = 0.046) and DC axons (*r* = −0.86, *P* = 0.01) and white matter demyelination with the density of F− SPMS ACST (*r* = −0.75, *P* = 0.022), LCST (*r* = −0.86, *P* = 0.004) and DC axons (*r* = −0.68, *P* = 0.048; Spearman correlation analysis). Please note that there was no difference between the proportion of F+ and F− SPMS cases who experienced a spinal relapse (*P* = 0.64, Fisher's exact test), the total number of spinal relapses (*P* = 0.32, Mann–Whitney test comparing the number of spinal relapses) or if the spinal relapse was seen early (within 2 years of diagnosis) or only later in disease (*P* = 0.99, Fisher's exact test).

### Microglia and macrophages in the spinal white and grey matter

**Figure 5:**
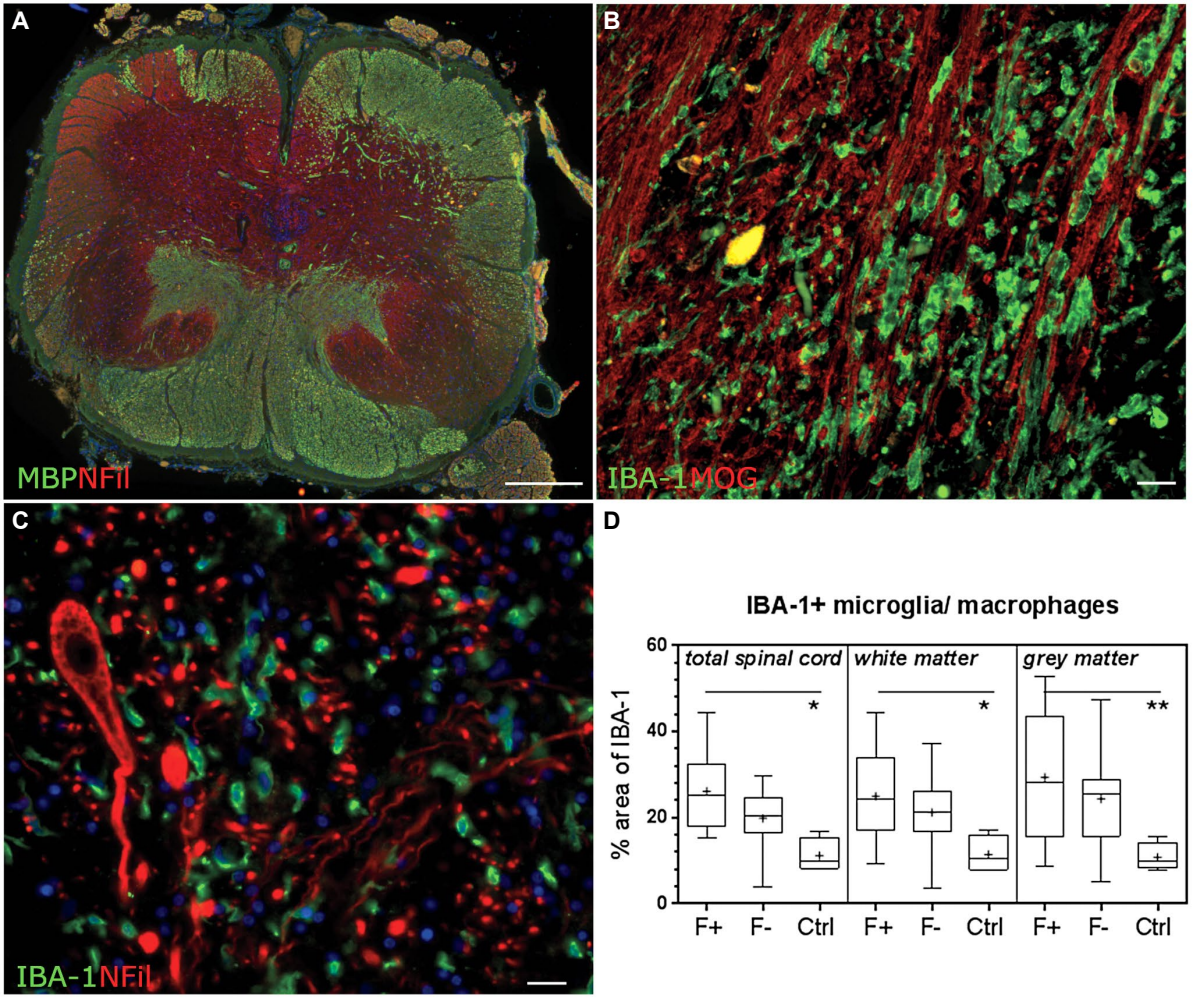
Microglia/macrophage activation in the SPMS cord. Example of demyelination in the case of F+ SPMS (**A**). Large numbers of IBA‐1+ microglia/macrophages (green) were present in the white matter parenchyma (**B**) and, although lower in number, in the grey matter (**C**; IBA‐1+ cells and neurofilament‐H+ neurons/neurites). The relative area of anti‐IBA‐1 immunoreactivity was increased in F+ SPMS in comparison to controls but was not different between F+ and F− SPMS cases (**D**). Data plotted as the box‐and‐whiskers plot showing the mean (+), median (line), interquartile range (box) and 5‐95 percentiles (whiskers) and compared by Kruskal–Wallis and Dunn's multiple comparison posttest. *P < 0.01. Scale bars: **A** = 1mm **B**, **C** = 40µm. Ctrl, control; F+, lymphoid‐like structure positive SPMS; F−, lymphoid‐like structure negative SPMS.

Microglial/macrophage activation was substantial in the demyelinated SPMS cord. IBA‐1+ microglia/macrophages were present in large numbers in white and grey matter of F+ and F− SPMS cases (Figure 5A–C), the majority of them with an activated morphology. The area of IBA‐1+ immunoreactivity in white, grey and the total cord was greater in F+ SPMS cases in comparison to control (F+ SPMS white matter = 25 ± 4%, ctrl = 11 ± 2%, *P* = 0.03; F+ SPMS GM = 29 ± 6%, ctrl = 11 ± 1%, *P* = 0.04; F+ SPMS total section = 26 ± 3%, ctrl = 11 ± 2%, *P* = 0.008) but was not different between F+ and F− SPMS, or between F− SPMS and control (Figure 5D).

### B cell infiltration in the meninges correlates with the extent of spinal cord pathology

We next investigated the interrelationship between the density of B and T cells with meningeal and perivascular inflammation of the underlying white matter (Table 3), with the extent of demyelination (Table 4) and relative axonal loss (Table 5). The correlation matrices demonstrate the statistically significant relationships found between the densities of the different lymphocyte populations quantified in the meninges and highlight the close association seen between meningeal inflammation (CD4, CD8 and CD20+ cells) and the perivascular lymphocytic infiltrates of the white matter. Correlation analysis of the SPMS groups revealed that it was only in the F+ SPMS cohort that the density of meningeal B cells correlated modestly (*r* = 0.684) with the extent of white matter demyelination (Table 4). The density of meningeal B cells in F+ SPMS also correlated with the extent of axon loss in the lateral corticospinal tract, dorsal column and combined spinal cord tracts axons (Table 5). The density of CD4 and CD8 cells did not correlate with the extent of demyelination or axon loss in F+ SPMS. The same pathological relationships between the number of meningeal B cells and demyelination and axonal loss were not evident in the F− SPMS cases, suggesting a less prominent role for B cell immunity in these cases (Tables 3, 4, 5). Previous work has reported a correlation between the density of CD3 meningeal infiltrates and axon loss (2). In our study, the sum of both CD4+ and CD8+ cells in all cases of SPMS was modestly correlated with axon loss (spearman *r* = −0.372, *P* = 0.0328), but this correlation did not exist in the separate F+ or F− SPMS groups.

**Table 3:** Meningeal inflammation and tissue infiltrates.

	Meninges			Perivascular white matter			
	CD8	CD4	Total	CD20	CD8	CD4	Total
CD20	*r* = 0.356 *P* = 0.176	*r* = 0.559 *P* = 0.027	*r* = 0.677 *P* = 0.005	*r* = 0.585 *P* = 0.005	*r* = 0.047 *P* = 0.865	*r* = 0.532 *P* = 0.036	*r* = 0.462 *P* = 0.074
CD8	–	*r* = 0.620 *P* = 0.009	*r* = 0.875 *P* < 0.000	*r* = 0.368 *P* = 0.147	*r* = 0.549 *P* = 0.024	*r* = 0.588 *P* = 0.015	*r* = 0.60 *P* = 0.013
CD4	–	–	*r* = 0.826 *P* < 0.000	*r* = 0.574 *P* = 0.018	*r* = 0.022 *P* = 0.936	*r* = 0.821 *P* < 0.001	*r* = 0.547 *P* = 0.025

Correlating meningeal B cells and T cells with inflammation in the spinal meninges and perivascular space. Total refers to the sum of CD20, CD4 and CD8+ cells. Spearman *r* and *P* values reported. Significant correlations highlighted.

**Table 4:** Meningeal inflammation and spinal cord demyelination in F+ SPMS.

		White matter lesion area	Grey matter lesion area	Total lesion area
F+ SPMS	CD20	*r* = −0.684 *P* = 0.034	*r* = −0.539 *P* = 0.114	*r* = −0.685 *P* = 0.035
	CD8	*r* = 0.143 *P* = 0.752	*r* = −0.132 *P* = 0.736	*r* = 0.048 *P* = 0.935
	CD4	*r* = 0.191 *P* = 0.665	*r* = 0.168 *P* = 0.693	*r* = −0.037 *P* = 0.930
	Total	*r* = 0.143 *P* = 0.752	*r* = 0.144 *P* = 0.736	*r* = 0.071 *P* = 0.882
F− SPMS	CD20	*r* = 0.349 *P* = 0.323	*r* = 0.325 *P* = 0.360	*r* = 0.249 *P* = 0.484
	CD8	*r* = 0.659 *P* = 0.092	*r* = 0.756 *P* = 0.041	*r* = 0.611 *P* = 0.118
	CD4	*r* = 0.366 *P* = 0.379	*r* = 0.146 *P* = 0.743	*r* = 0.144 *P* = 0.736
	Total	*r* = 0.658 *P* = 0.092	*r* = 0.659 *P* = 0.093	*r* = 0.539 *P* = 0.176

Correlating B cells and T cells with demyelination. Total refers to the sum of all lymphocytes. F+ SPMS = lymphoid‐like structure positive secondary progressive multiple sclerosis; F− SPMS = lymphoid‐like structure negative secondary progressive multiple sclerosis. Spearman *r* and *P* values reported. Significant correlations highlighted.

**Table 5:** Meningeal B cells and axon loss in F+ SPMS.

		A CST	L CST	D Col	All tracts
F+ SPMS	CD20	*r* = 0.714 *P* = 0.136	*r* = 0.893 *P* = 0.012	*r* = 0.886 *P* = 0.033	*r* = 0.684 *P* < 0.001
	CD8	*r* = 0.172 *P* = 0.782	*r* = 0.801 *P* = 0.098	*r* = −0.420 *P* = 0.482	*r* = 0.035 *P* = 0.903
	CD4	*r* = −0.208 *P* = 0.737	*r* = 0.439 *P* = 0.193	*r* = −0.542 *P* = 0.346	*r* = −0.189 *P* = 0.499
	Total	*r* = 0.119 *P* = 0.848	*r* = 0.762 *P* = 0.134	*r* = −0.357 *P* = 0.555	*r* = 0.120 *P* = 0.680
F− SPMS	CD20	*r* = −0.029 *P* > 0.999	*r* = −0.028 *P* = 0.989	*r* = 0.046 *P* = 0.931	*r* = −0.066 *P* = 0.795
	CD8	*r* = −0.429 *P* = 0.419	*r* = −0.371 *P* = 0.497	*r* = −0.370 *P* = 0.470	*r* = −0.179 *P* = 0.478
	CD4	*r* = −0.543 *P* = 0.297	*r* = −0.314 *P* = 0.564	*r* = −0.553 *P* = 0.255	*r* = −0.379 *P* = 0.121
	Total	*r* = −0.314 *P* = 0.564	*r* = −0.486 *P* = 0.356	*r* = −0.423 *P* = 0.398	*r* = −0.166 *P* = 0.510

Correlating B cells, T cell and total B and T cells with axon loss in the anterior corticospinal tract (A CST), lateral corticospinal tract (L CST), dorsal column (D Col) or with the mean axon loss across all assessed tracts. F+ SPMS = lymphoid‐like structure positive secondary progressive multiple sclerosis; F− SPMS = lymphoid‐like structure negative secondary progressive multiple sclerosis. Spearman *r* and *P* values reported. Significant correlations highlighted.

## Discussion

It is now well established that leptomeningeal inflammation is a prominent feature of cortical pathology and strongly associates with the extent of subpial neocortical grey matter demyelination, microglial activation and neuronal loss in a substantial proportion of acute and progressive MS cases at post‐mortem (6, 24, 35, 39). Here, we report that spinal cord pathology is also more severe in SPMS cases characterized by elevated leptomeningeal inflammation of the forebrain. The more severe cord pathology was characterized by a greater degree of lymphocyte infiltration of the spinal leptomeninges, perivascular spaces and parenchyma, microglial activation and axonal loss, which correlated with the density of B cell, but not T cell, infiltrates in the spinal meninges. This work adds to our knowledge of the importance of CNS‐resident B cells to the pathology of progressive disease and furthers our understanding of the importance of the compartmentalized meningeal inflammatory response to tissue damage in progressive MS.

### Meningeal inflammation and lymphoid‐like structures are widespread and not only restricted to the brain meninges in progressive MS

Screening of three spinal segments per case, representing cervical, thoracic and lumbar cord, revealed that three out of the 11 MS cases that were characterized by lymphoid‐like structures in the cerebral meninges had similar structures in the spinal cord meninges. In the brain, where we first observed lymphoid‐like structures, it was often necessary to screen twelve or more blocks of cortical/subcortical tissue before such infiltrates were noted, with 5‐67% of the tissue blocks screened per case found to contain lymphoid‐like structures (1, 24). Therefore, it is likely that a more extensive screening along the entire length of the cord might have revealed more cases with evidence of lymphoid‐like structures. However, the F+ SPMS cases in which we did not find lymphoid‐like structures in the spinal cord still exhibited extensive meningeal inflammation, including the presence of immune cell aggregates rich in B cells. The fact that the immune cells in the cord meninges were not always organized into lymphoid‐like structures may reflect the different anatomical structure (only a single sulcus at the anterior median fissure) of the spinal cord, which may not be as conducive to the accumulation of large and organized immune cell infiltrates. Large, organized lymphoid‐like structures were also not seen in the cerebellum, a structure with very limited sulcal volume, and where, as in this study, increased meningeal inflammation associated with a more severe subpial pathology (25).

### Meningeal inflammation is associated with elevated myelin and axonal pathology

The current study suggests that the presence of lymphoid‐like structures in the forebrain signifies a globally elevated inflammatory pathology, as SPMS cases with lymphoid‐like structures in the brain showed extensive demyelination and axonal loss in the spinal cord. The extensive demyelination and axon loss in the cord is in line with that reported by others (14, 19, 48). Demyelination was highly variable and was not different between F+ SPMS and F− SPMS cases (which is likely to be the result of the relatively small number of cases being studied, due to the limited available spinal cord from these previously characterized cases). Of note, and evident in both SPMS cohorts, was the finding that the demyelinated spinal grey matter covered a greater area than the demyelinated white matter in the same case. These findings suggest that immune‐mediated damage arising from the overlying meninges is only one component of the pathogenetic mechanisms, and that other local and distal indirect contributions from cells in the parenchyma also contribute to lesion development and expansion. Our data also shows that it is not only subpial grey matter lesions which can topographically associate with meningeal inflammation and supports previous neuroimaging descriptions of gradients of white, as well as GM pathology, greatest near the brain's surfaces, in the central white and grey matter areas (8, 41, 51). It would be interesting to determine if gradients of spinal cord tissue damage exist in the subpial white matter or in the grey matter away from the central canal.

Axonal damage is a major contributor to clinical progression in MS (7, 33, 48, 56). Our finding of an increased axon loss in F+ compared to F− SPMS cases most likely reflects the increased meningeal and parenchymal inflammation and demyelination in this group of patients. Myelin loss strips axons of crucial metabolic and structural support, and increased inflammation, be it focal inflammatory infiltrates or more diffuse inflammation of the tissue parenchyma and meninges, will sustain a neurotoxic and damaging environment (17, 46, 55). Axon loss in the spinal cord does not associate with the spinal cord cross‐sectional area (48). This finding indicates caution when interpreting magnetic resonance imaging measures of cord atrophy as a surrogate for neuroaxonal loss in the patient. A potential component of this dissociation between axon density and cord cross‐sectional area will include cellular infiltration and tissue inflammation, reactive microglial and astrogliosis, which are likely to be quite extensive in cases harboring lymphoid‐like structures. By improving our understanding of the associations that exist between meningeal inflammation and spinal cord tissue pathology in some cases might help in the interpretation of new and powerful *in vivo* imaging technologies (21, 53).

### B cells and not T cells are most strongly associated with spinal cord pathology

The only immune cell component of the cord meninges that was consistently different between the F+ and F− SPMS groups was the number of CD20+ B cells, which were 3‐fold higher in number in the F+ SPMS cases. The density of CD8+ T cells was greater than that of CD4+ T cells in all measured compartments, in line with previous findings (2, 12). However, the number of CD8 and CD4+ T cells varied considerably and did not differ between F+ and F− SPMS groups.

**Figure 6:**
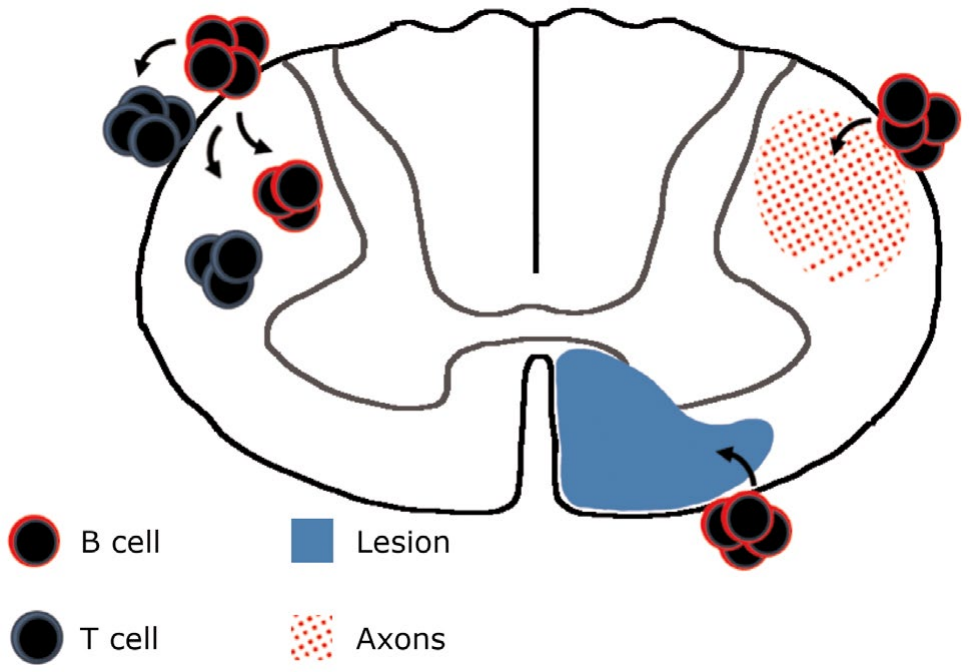
The main findings of this study are highlighted. Meningeal inflammation and particularly B cell inflammation (red circles), is associated with greater CD4+ T cell (blue circles) and B cells in the connective tissue and cord parenchyma. CD20+ B cell density of the meninges also correlated with the extent of underlying white matter demyelination (blue sector) and axon loss (red sector). These correlations only existed in the F+ SPMS cohort characterized in the presence of lymphoid‐like structures in the forebrain meninges.

It has been shown previously that the density of meningeal CD3+ T cells correlated with axon loss in the normal‐appearing cervical spinal cord (2), whilst the number of CD8+ T cells correlated with a loss of small caliber axons in the lumbar‐sacral cord (12). Whilst neither the number of CD4+ or CD8+ T cells correlated with axon loss in our study, we saw a significant association between the total number of T cells (the sum of CD4 and CD8 cell densities) and axon loss, which supports the work of others. This modest correlation did not exist in our subgroup analysis, where it was only the density of CD20+ B cells in the F+ SPMS cohort that associated with measures of parenchymal pathology (see Figure 6).

In F+ SPMS, CD20+ B cells were often found as aggregates in the spinal cord meninges, which associated with increased numbers of CD4+ T cells in the meninges and perivascular spaces and with increased numbers of activated microglia/macrophages, and increased demyelination and axon loss (Tables 3, 4, 5 and Figure 6). Activated memory B cell subsets from MS can be stimulated to secrete elevated levels of TNF, LTα, IL6 and GM‐CSF, and are highly effective antigen‐presenting cells, both in the inflamed organ and draining lymph tissues (29). GM‐CSF secreted by B cells activates myeloid cells that worsen experimental disease, whilst GM‐CSF and IL6 secreting B cells are more abundant in MS and are at least in part ablated by anti‐B cell therapy and indicate a successful response to therapy (5, 30). New, emerging B cells, which repopulate the immune system following anti‐CD20 treatment, lack such a pro‐inflammatory profile (15, 30). Ex‐vivo activation of B cells stimulates CD4 and CD8 proliferation and pro‐inflammatory activation, whilst B cell depletion diminishes the capacity of CD4+ cells from MS patients to be stimulated through an MHC II/HLA‐DR15 B cell‐to‐ CD4+ T cell interaction (4, 26, 42). These stimulated effector CD4+ cells have CNS‐homing and auto‐reactive phenotype to antigens expressed in the MS neocortex (26). Genetic factors impact the risk of MS but their relative contribution to disease outcome is still to be defined. HLA‐DR15 status is the principal genetic susceptibility locus for MS, whilst also being associated with elevated forebrain and spinal cord pathology, albeit more strongly linked to T cell, rather than B cell, inflammation (12, 13, 58). Alongside a central role in antigen presentation and T cell activation, effector memory B cells from MS patients can directly induce oligodendrocyte and neuroaxonal injury *in vitro* (31, 32) and suggests that ablating this B cell central memory pool could be important in mediating a disease‐modifying effect (3).

We suggest that a higher level of demyelination and axonal loss in MS cases expressing meningeal lymphoid‐like structures may in part be related to the increased immune cell infiltration in the cord meninges, which via the production of pro‐inflammatory cytokines and chemokines, including TNF, IL6, IFNγ and CXCL13, could cause direct and/or indirect damage to the underlying tissues (18, 28, 37, 38). This inflammatory milieu is reflected in the CSF (27) and a small number of inflammatory mediators, including B cell chemokines, define both a post‐mortem and a clinical cohort with a higher lesion load and poor prognosis (38). Since CSF circulates through the ventricles, over the brain and in and over the cord, our results reinforce the idea that an inflammatory milieu present throughout the CSF space in a large proportion of MS cases from early stages and during the progressive phase, could lead to diffuse tissue inflammation, activation of astroglia and disruption of the glial limiting barriers of the pia and parenchyma.

### Limitations of this study

The current study has involved a small number of cord samples, which was due to the limited availability of appropriate samples from previously characterized cases of F+ and F− SPMS. The relative infrequent finding of *bona fide* lymphoid‐like structures may well reflect the need to screen more extensively along the length of the cord to better understand the extent of the spinal leptomeningeal inflammation. We did not quantify the density of macrophages or plasma cells in this study, cells which are found at elevated numbers in cases that follow an active progressive disease (17, 24, 36) and may also associate with the compartmentalized immune response and topographically with subpial pathology in other brain areas (28). No association was seen between F+ status and the incidence of spinal relapses. Although documentation of relapses is unreliable (50), the UK MS Tissue Bank reports include general practitioner and/or hospital recorded relapses.

## Conclusions

The extent of spinal cord atrophy and demyelination is an early clinical and imaging prognostic of risk to clinically definite MS and of later disease severity (9, 47). For these reasons we need to know more about mechanisms that contribute to the damage of the spinal cord if we are to identify surrogate fluid markers or to further our understanding to aid the development of imaging technologies. In this report, we further corroborate the hypothesis that B cells play a key role in MS pathology and highlight the association between B cell inflammation in the forebrain and that seen in the spinal cord and between B cell inflammation of the spinal meninges and lymphocyte infiltrates, demyelination and axon loss in clinically eloquent pathways.

## Conflict of Interest

RR has received speaking honoraria from Roche, Novartis and ECTRIMS and grant funding from MedImmune plc. OWH has received travel reimbursement or speaking honoria (paid to Swansea University) from Roche, the Neurology Academy and ECTRIMS. All other authors have no relevant disclosures.

## Author Contributions

CR, RM, OWH and RR designed the study. CR, RM, FR and RN performed the experiments and CR, OWH and RR performed the analysis and drafted the manuscript. All authors contributed to the writing of the submitted document.

## Data Availability

The data that support the findings of this study are available from the corresponding author upon reasonable request.
